# Determinants of systemic hypertension in older adults in Africa: a systematic review

**DOI:** 10.1186/s12872-019-1147-7

**Published:** 2019-07-22

**Authors:** William Kofi Bosu, Justice Moses Kwaku Aheto, Eugenio Zucchelli, Siobhan Theresa Reilly

**Affiliations:** 1 Department of Public Health and Research, West African Health Organisation, Bobo-Dioulasso, 01 BP 153 Burkina Faso; 2 Division of Health Research, Faculty of Health & Medicine, Furness Building, Lancaster University, Lancaster, LA1 4YG UK; 3 Department of Biostatistics, School of Public Health, University of Ghana, PO Box LG 13, Legon, Accra Ghana; 4 Madrid Institute for Advanced Study (MIAS), Universidad Autonoma de Madrid, C/ Einstein, 13 Pabellón C 1a planta, E-28049 Madrid, Spain

**Keywords:** Hypertension, Determinants, Risk factors, Older adults, Systematic review, Multivariate analysis, Africa

## Abstract

**Background:**

An estimated 55% of older adults in Africa have systemic hypertension, a major risk factor for stroke, heart failure and dementia in the region. The risk factors associated with hypertension in this population group in Africa have not been systematically evaluated. We, therefore, undertook a systematic review to identify these risk factors.

**Methods:**

We searched for population-based studies of adults aged ≥50 years living in Africa and reporting an estimate of hypertension and associated risk factors. We included articles published in any language between January 1980 and May 2018 using a comprehensive search strategy. We extracted data including the sample characteristics, prevalence of hypertension and risk factors with their effect sizes.

**Results:**

From an initial 10,719 records, we retained 63 eligible full text articles for review out of which we analyzed 23 studies made up of 19 primary and four multiple publications which had data on risk factors from bivariate or multivariable analysis. The primary studies, published from 2010 to 2018, involved a total of 30,500 participants in 12 different countries with mean ages ranging from 62.7 ± 9 years to 76.9 ± 8.4 years. Through narrative synthesis, we found consistent determinants of hypertension (overweight/obesity and history of stroke), less consistent but frequent determinants (including older age group, female sex and urban residence), inconsistent determinants (including education, wealth index, alcohol intake and physical activity) and nonsignificant covariates (marital status and having health insurance). Overall, the highest adjusted odds ratios were those associated with obesity and history of stroke.

**Conclusion:**

The key determinants of systemic hypertension in older adults in Africa are older age group, overweight/obesity, history of stroke and female sex. Health programmes should promote weight reduction throughout the life course, including during the middle and older age of African adults.

**Electronic supplementary material:**

The online version of this article (10.1186/s12872-019-1147-7) contains supplementary material, which is available to authorized users.

## Background

Africa experiences a significant burden of cardiovascular diseases, although this is often obscured by the greater priority to and interest in infectious diseases. According to the Global Burden of Disease Study, cardiovascular diseases were the leading cause of death in Africa in 2017, being responsible for 1.42 million deaths in that year or 16.4% of the total deaths in all ages compared with 11.3% of total deaths in 1990 [1]. The mortality represents a 61.0% increase over the estimated number of cardiovascular deaths in 1990. High systolic blood pressure (SBP) accounted for nearly two-thirds of the cardiovascular deaths in Africa in 2017. The region has the highest prevalence of raised blood pressure (27%) in the world [2]. The increasing burden is attributed to ageing, increasing population and adoption of unhealthy lifestyles. The weak health systems, low literacy levels, infrequent medical check-ups and prevailing poverty contribute to frequent misconceptions about hypertension, low detection and poor control of the disease.

Africa, therefore, has an onerous task in meeting the global target of 25% relative reduction in the prevalence of hypertension by the year 2025. National responses to noncommunicable diseases (NCDs) have been slow, with many countries lacking diet and physical activity policies [3]. In 2015, only 25.8% of African countries had developed or adopted clinical practice guidelines for the management of hypertension [4]. On the other hand, the number of African countries that have conducted the chronic disease risk factor STEPS surveillance has doubled from 20 in 2009. There is a renewed political commitment to tackle NCDs following the third high-level meeting of the United Nations General Assembly in October 2018 during which Heads of State and Governments committed to reorienting health systems to respond to the needs of the rapidly ageing population in relation to NCDs [5].

Individual studies observe that older age, high body mass index (BMI), alcohol use, female sex and urban residence are among the main factors associated with hypertension in adults [6–9]. However, there have been few systematic reviews of the determinants of hypertension in Africa [10]. Our systematic review among workers in West Africa found that the determinants included male sex, older age group, higher socioeconomic status, obesity, alcohol consumption, plasma glucose, and sodium excretion [11]. There is currently no published report of a systematic review of the determinants of hypertension among older adults in Africa. It is in this context that, we systematically reviewed the literature to identify factors associated with hypertension in older adults in Africa.

## Methods

This study was undertaken as part of a systematic review whose protocol has been published earlier [12]. The study population, search terms and the meta-analysis have been described elsewhere [13]. The reporting of our findings follows the Preferred Reporting Items for Systematic Reviews and Meta-Analyses (PRISMA) guidelines [14]. The methods described here focus on the risk factors associated with hypertension.

### Data sources and search strategy

**Fig. 1:**
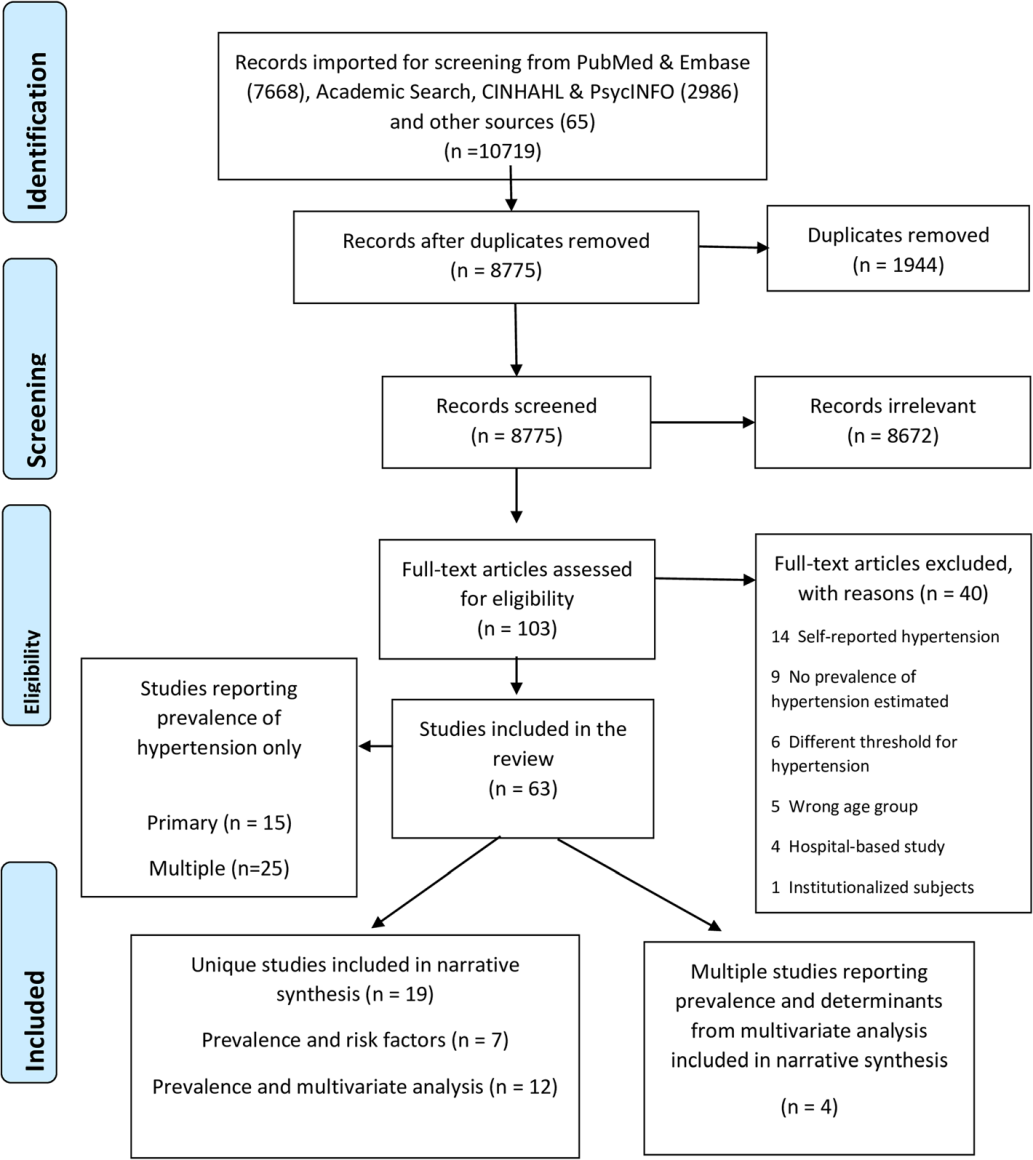
Flow chart of study selection

We searched major electronic databases, Medline and Embase through Ovid, PsychInfo and CINAHL as well as the African Journals Online repository for articles published between 1 January 1980 to 28 May 2018. We also searched grey literature via ProQuest and Google scholar. Guided by the Population, Intervention, Comparison, and Outcome (PICO)-strategy, we used search terms designed to comprehensively identify potential studies in which the prevalence of hypertension had been reported. The search terms included those related to hypertension or blood pressure combined with the names of each of the 58 African countries (See Additional file 1 Table S1). The identified articles were screened at the title, abstract and full-text levels (Fig. 1). The bibliographies of included studies were also searched to identify additional studies.

The included studies were limited to those on human subjects but there was no language restriction. We took account of multiple publications on the same study sample and retained the one that was most informative in the analysis (in order to avoid multiple counts). Where necessary, we sought supplementary information from the other multiple publications. Longitudinal studies which reported the prevalence of hypertension with associated risk factors at baseline and at the end of follow-up were considered as one study which contributed two data points. Similarly, if a study conducted in more than one African country, say in country A and country B, reported the prevalence of hypertension in each country along with associated risk factors, then it was considered as one study contributing two data points.

### Study selection and data extraction

The included studies were population-based cross-sectional or follow-up designs conducted among subjects aged 50 years or older living in Africa. We included studies that reported the prevalence or incidence of systemic hypertension along with associated risk factors or correlates. We excluded studies conducted among patients in hospital, residents of restricted institutions or migrants living outside Africa or studies reporting self-reported or non-systemic hypertension.

We used the Covidence software to manage the selection of studies [15]. This programme removed identical studies from different databases and also guided the independent evaluation of potential studies on their conformity with the inclusion criteria by two reviewers (WKB, JMKA). Any discrepancies between the two reviewers were resolved through consensus.

After assigning each included study a unique identification number, we extracted data on the publication characteristics, sociodemographic characteristics of the subjects, main objectives, sampling technique, sample size, anthropometric measurements, method of blood pressure measurements, and the prevalence or incidence of hypertension on to a pre-designed form in Excel. We also extracted age- and sex-specific prevalence of hypertension (where reported) as well as the crude and adjusted effect sizes from multivariable analysis, including the 95% confidence intervals (CI). The latter data on independent correlates of hypertension from multivariable analysis were obtained not only from primary studies, but also, from multiple publications. These multiple publications, which could be single- or multi-country studies, were all studies that had been published as and captured in the included primary studies.

### Data synthesis

We classified included studies into three groups: i) single publications reporting the prevalence and some associated risk factors such as age, sex or BMI; ii) single publications reporting prevalence and the determinants from multivariate analysis; and iii) multiple publications reporting determinants from multivariate analysis. We excluded studies which reported only the prevalence of hypertension (without further analysis) from the synthesis.

The risk factors or determinants were classified into demographic, socioeconomic, lifestyle and co-morbidity groups and analyzed through narrative synthesis. The demographic variables evaluated in the different studies included age, sex, marital status, residence, race or tribe and religion in different combinations. The socioeconomic variables included educational level, occupation, wealth quintile or income, and membership of a health insurance scheme. Lifestyle variables modelled included alcohol intake, smoking, fruit or vegetable intake, physical activity and BMI status. The final group of covariates were comorbidities including history of diabetes or stroke, self-reported chronic illness, disability or depression.

For each risk factor, we identified the reference category and considered the effect size (odds ratio, prevalence ratio, relative risk) as harmful if it was greater than 1 and protective if it was lower than 1. We identified the harmful, protective, statistically significant and non-significant determinants from both crude (bivariate) and multivariate analyses. All included studies set the *p* value for their tests of significance at 5%. We looked for consistency in the direction and patterns of association of hypertension across the studies.

### Quality appraisal of studies

Two reviewers (WKB, JMKA) independently assessed the quality of the included primary studies using a tool developed by Hoy and others [16] and validated specifically for prevalence studies. This appraisal tool uses ten questions to assess both external validity (e.g. representativeness of the sample, participation rate and sampling methods) and internal validity (e.g. direct data collection from subjects or from proxy, suitability of case definition, reliability of study instrument, application of same measurement methods for all subjects and appropriateness of exposure period) in each study. Based on an overall judgement from these criteria, we classified each study as having a low, moderate and high risk of bias. Our decision to include studies rated as high risk of bias our narrative synthesis of risk factors was guided by the results of a sensitivity analysis conducted to evaluate the effect of their exclusion on a pooled estimate of the prevalence of hypertension [13].

## Results

### Description of selected studies

We screened 8,775 citations after removing duplicates out of which we retrieved 103 full-text versions to assess their eligibility for inclusion (Fig. 1). Of the latter, 63 studies, made up of 34 primary studies and 29 multiple publications, met the inclusion criteria for review. However, 40 of them reported information on only the prevalence of hypertension and so were excluded from further analysis. Of the remaining 23 studies, seven provided information on risk factors associated with the prevalence while 16 made up of 12 primary and four multiple publications provided data on the determinants of hypertension from multivariate analyses.

One of the seven primary publications reported sex-specific prevalence of hypertension in cities in two African countries (Bangui and Brazzaville) and so provided two data contributions relating to hypertension and its associated risk factors in two countries [17]. Of the four multiple publications, three were multi-country publications based on the Study on Global Ageing and Adult Health (SAGE) in Ghana and South Africa, and so these provided a combined total of six data contributions [18–20]. The fourth multiple paper, based on SAGE Ghana, reported determinants separately for systolic hypertension (SHTN) and diastolic hypertension (DHTN) [21]. The two respective primary publications for the SAGE Ghana and South African studies were included in the 12 unique studies providing data on determinants of hypertension [22, 23]. Thus, there were 19 primary (providing 21 data points) and four multiple (non-primary) publications (providing eight data points) [18–21] of relevance that were analyzed.

Based on the Hoy et al. tool, we judged that 16 (84.2%) of the 19 included primary studies had either a low (63.2%) or moderate risk (21.0%) of bias (Table 1; Additional file 2 Table S2). Three (15.8%) studies were considered to have a high risk of bias, usually because of use of non-representative samples or low participation rate. We retained these studies in the narrative synthesis because we found in a sensitivity analysis conducted as part of a meta-analysis that, excluding them did not have any significant influence on the estimated prevalence of hypertension [13].

**Table 1:** Sociodemographic characteristics of study sample and sex-specific prevalence of hypertension in included primary articles

Primary Reference	Country	Study year	Type of participants	% Participation rate of eligible sample	% Female	% urban	% aged 70+ years	Mean age ± sd	% currently married	% no education	Number whose hypertension status assessed	% males with hypertension	% females with hypertension	% total subjects with hypertension	Overall risk of study bias
Abegunde 2013 [24]	Nigeria		adults aged ≥60 years	98.4	61.1	50.2	57.9	72.2 ± 9.5 (urban), 70.8 ± 8.1 (rural)	63.0	93.8	600			36.5	Low
Dewhurst 2013 [25]	Tanzania	2010	Rural cohort aged ≥70 years (DSS)	93.5	56.3	0.0	100.0				2223	62.2	75.8	69.9	Low
Guerchet 2012A [17]	CAR	2009	adults aged ≥65 years	96.3	58.1	100.0	59.9	72.6 ± 6.1	41.6		461	47.2	62.7	56.2	Low
Guerchet 2012B [17]	Congo	2009	adults aged ≥65 years	96.3	61.9	100.0	72.0	74.4 ± 6.7	38.1		515	66.3	76.2	72.4	Low
Hammami 2011 [26]	Tunisia	2009	Elderly aged ≥65 years	100	66.2	86.3	62.0	72.3 ± 7.4	60.0	76.9	598	45.0	55.6	52.0	Low
Hien 2014 [27]	Burkina Faso	2012	Elderly aged ≥60 years	100	44.7	100.0	39.3	69 ± 7	58.7	54.1	389	79.1	86.2	82.3	Low
Iribhogbe 2013 [28]	Nigeria	2012	church attendants aged ≥50 years	100.0	50.0		23.5	67.55 ± 16.61			200	58.0	35.0	46.5	High
Koopman 2012 [29]	Ghana	2010	registered inhabitants ≥50 years	85.4	48.1		35.3				924	25.6	22.5	24.1	Low
Macia 2012 [30]	Senegal	2009	adults aged ≥50 years	NR	47.4	100.0	19.2		74.4	45.6	500	63.9	67.1	65.4	Moderate
Mathenge 2010 [31]	Kenya	2008	adults aged ≥50 years	87.7	52.3	32.7	26.4			33.1	4396			50.1	Low
Minicuci 2014 [22]	Ghana	2008	adults aged ≥50 years	95.9	50.3	40.6	32.5		59.8	54.0	4724	51.5	50.7	51.1	Low
Mkhize 2011 [32]	South Africa	2008	elderly persons aged ≥60 years		83.0						270	82.6	91.5	90.0	Low
Mugisha 2013 [33]	Uganda	2013	rural adults aged ≥50 years	72.3	58.2	0.0	27.7	median 62	48.5	28.0	1449	36.5	38.3	37.5	Moderate
Nuertey 2017 [34]	Ghana	2014	registered members of the national pensioners association	93.0	31.4		28.5		73.0	12.1	4439			47.8	High
Osman 2017 [35]	Ghana	2016	free-living adults aged ≥65 years	100.0	66.3				46.0	51.0	400	57.8	52.5	54.3	High
Peltzer 2013 [23]	South Africa	2008	adults aged ≥50 years	60	57.3	66.7	23.8		53.3	25.4	3672	74.4	79.6	77.4	Moderate
Pilleron 2017A [36]	CAR	2012	adults aged ≥65 years	94.7	62.2	51.5	60.4	72.7 ± 6.4	34.3		967			53.7	Low
Pilleron 2017B [36]	Congo	2012	adults aged ≥65 years	94.7	60.9	48.7	66.8	73.5 ± 6.7	38.8		1023			68.0	Low
Raji 2017 [37]	Nigeria	2007	elderly cohort aged ≥65 years	76.8	55.1	37.4	79.0	76.9 ± 8.4	55.7	44.8	1469	62.0	70.7	66.8	Moderate
Scholten 2011 [38]	Uganda	2010	adults aged ≥50 years	> 99%	61.2		34.9		32.4	24.0	510	30.3	34.6	32.9	Low
Tianyi 2017 [39]	Cameroon	2013	rural adults aged ≥50 years		68.9	0.0	24.2	62.7 ± 9	89.4	82.0	501	60.9	55.7	57.3	Low

*CAR* Central African Republic

### Description of sample characteristics

The 19 primary studies / publications covered 12 different countries with four from Ghana [22, 29, 34, 35]; three from Nigeria [24, 28, 37]; two each from Central African Republic/Congo [17, 36], South Africa [23, 32] and Uganda [33, 38]; and one each from Burkina Faso [27], Cameroon [39], Kenya [31], Senegal [30], Tanzania [40] and Tunisia [26] (Table 1). They involved a total of 30,500 participants out of whom 30,230 had their blood pressure measured. The prevalence of hypertension ranged from 24.1% in a rural community in northern Ghana [29] to 90.0% in a South African township [32]. The crude prevalence across all the subjects in the 21 data contributions was 55.9%.

The study participants were diverse and included older adults living generally in the community, those enrolled in cohort studies or a demographic surveillance site, church attendees or registered members of pensions associations (Table 1). Their ages ranged from 50 to 110 years. Their mean ages, reported from ten data points(nine studies) ranged from 62.7 ± 9 years in Cameroon [39] to 76.9 ± 8.4 years in Nigeria [37]. Across 19 data points (18 studies), the proportion of subjects aged ≥70 years varied from 19.2% in Dakar, Senegal [30] to 100.0% in the rural Hai district of Tanzania [25].

Overall, 51.9% of the enrolled subjects across the studies were female. In individual studies, the percentage female ranged from 31.4% [34] to 83.0% [41] (Table 1). Only four studies enrolled predominantly male subjects [27, 29, 30, 34]. The proportion of enrolled subjects who were currently married ranged from 32.4% [38] to 89.4% [39] (Table 1). In the Central African Republic, 38–42% of enrolled subjects with a mean age of about 73–47 years were currently married [17, 36]. In contrast, in Tunisia and Nigeria where the participants were slightly younger with a mean age of 72 years, 60–63% of them were currently married [24, 26]. The proportion of subjects with no formal education ranged from 12.1% among registered pensioners in Ghana [34] to 93.8% among elderly subjects in Nigeria [24] in 13 studies (13 data points) in which it was reported (Table 1). Except for one cohort study in Nigeria [37], all the studies were cross-sectional in design.

The study setting was rural in four studies (four data contributions), urban in six studies (seven data contributions) and mixed in nine studies (ten data contributions). The mixed settings were predominantly rural [22, 31, 36, 37] or urban [23, 24, 36]. Enrolled subjects were under demographic surveillance in Ghana [29], Tanzania [40] and Uganda [33, 38]. The proportion of participants who were overweight or obese, based on nine studies (11 data points), varied 100-fold from 0.8% of subjects in rural Ghana [29] to 80.0% of subjects in urban Tunisia [26]. The included studies were published from 2010 to 2018 with 2013 as the median year of publication.

#### Risk factors for hypertension

Except for one study which used a multilevel multinomial logit model [21], all the 16 studies providing 20 data points which investigated the determinants of hypertension did so using standard logistic regressions. The studies were conducted between 2007 and 2016 and published between 2010 and 2017. The number of covariates evaluated for their association with the binomial outcome of hypertension in the models ranged from five [19, 30] to thirteen [20]. One study provided separate estimates for the prevalence of hypertension in two countries, Central African Republic (CAR) and the Congo [36]. However, in the evaluation of factors associated with hypertension, the two countries were included in the same models.

##### Demographic factors

***Age and sex.*** The age-specific prevalence of hypertension was available, to varying degrees, in only eight studies (nine data contributions) in tables and in a graph (Table 2). From the limited data, we observed that the prevalence in the oldest age group was generally higher than that of the youngest age group in CAR, Congo, Ghana, Nigeria, South Africa and Tanzania [25, 35–37, 42], the exceptions being the studies in Cameroon [39], Ghana [22] and Tunisia [26]. The widest differences of more than ten percentage points between these extreme age groups were observed in Ghana [35], Kenya [31] and CAR [36]. In the studies in which the prevalence of hypertension was reported in three or more age groups, there was rarely a monotonic increase in prevalence with increasing age. The prevalence often peaked in the middle age group(s). However, there was a steady increase in the prevalence with age in Ghana [35] and among urban and rural men and women in Kenya [31].

**Table 2:** Age-specific prevalence of hypertension in studies in older adults in Africa

No.	Reference	Country	50–59	60–69	70–79	80 +	50–59	60–69	70–79	80 +	50–59	60–69	70–79	80 +	50–64	65–74	75+	65–69	70–74	75–79	80 +	65–74	75–84	85+
			M	M	M	M	F	F	F	F	T	T	T	T	T	T	T	T	T	T	T	T	T	T
1	Dewhurst 2013 [25]	Tanzania			60.6	65.9			75.0	77.3			68.6	72.5										
2	Hammami 2011 [26]	Tunisia										51.5	54.9	45.8										
3	Minicuci 2014 [22]														50.0	54.2	49.9							
4	Osman 2017 [35]	Ghana													52.0	54.5	63.5					52.0	54.5	63.5
5	Phaswana-Mafuya 2013 [42]	South Africa									74.9	80.6	78.4											
6	Pilleron 2017a [36]	CAR																50.1	53.2	52.7	63.6			
7	Pilleron 2017b [36]	Congo																62.9	72.7	67.8	70.6			
8	Raji 2017 [37]	Nigeria																66.2	68.9	60.1	68.1			
9	Tianyi 2017 [39]	Cameroon	59.5	68.6	52.3	54.5	53.8	62.0	48.9	30.0	54.9	62.2	50.5	50.0										

*CAR* Central African Republic, *F* females, *M* males, *T* total subjects (both sexes)

The sex difference in the prevalence of hypertension within the age groups, available from two studies, was variable, being consistently higher in females in each age group in Tanzania [25] or lower in Cameroon [39] (Table 2). In individual studies, the prevalence of hypertension ranged from 25.6 to 82.6% in the men and from 22.5 to 91.5% in the women. Overall, the crude prevalence of hypertension in 10,478 females (61.6%) was higher than that of 8,327 males (56.9%) in the 15 studies (16 data contributions) in which the sex-specific prevalence was provided. In nine of these studies with ten data contributions, the prevalence was higher in females [17, 23, 25–27, 30, 32, 33, 37, 38] and in the remaining six, it was higher in males [22, 28, 29, 35, 39] (Table 1). The relationship between older age group or female sex and hypertension was statistically significant in Tunisia [26], South Africa [23], CAR and Congo [36] (Tables 3 and 4). That of older age group, but not sex, was statistically significant in Senegal [30] and in Ghana [22] while that of sex but not older age group was statistically significant in Nigeria [37].

**Table 3:** Overview of risk factors associated with hypertension among older adults in Africa

No.	Country	Reference	Significant harmful (higher HTN) variables in crude analysis	Significant protective (lower HTN) variables in crude analysis	NS variables in crude analysis	Harmful Determinants of (higher) HTN	Determinants of lower HTN	NS variables in multivariate model
1	Nigeria	Abegunde 2013 [24]				Female gender, decreasing monthly income, increasing BMI		alcohol intake
2	Ghana	Boateng 2017 [21]				For systolic stage 1 or 2 HTN: increasing BMI category, alcohol intake, higher wealth index, female sex	traditional religion,	Place of residence, depression or ethnicity for stage 1 or 2 SHTN; religion not significantly associated with stage 1 SHTN.
3	Ghana	Boateng 2017 [21]				For diastolic stage 1 or 2 HTN: increasing BMI category, higher wealth index	traditional religion, other religion	Place of residence, depression or ethnicity for stage 1 or 2 DHTN; religion except traditional or other religion not significantly associated with stage of DHTN.
4	Tanzania	Dewhurst 2013 [25]				BMI (continuous variable), female sex, older age group ≥85 years, Chagga tribal origin, upland village dwelling		Presence of (moderate or severe) disability (by Barthel Index Score); age groups 75–79 and 80–84 years
5	Tunisia	Hammami 2011 [26]	Females, Older age group, dependency, self-reported diabetes, overweight, abdominal obesity		Education, urban-rural residence, depression, marital status, physical activity	Diabetes, BMI, Dependency (disability)		age, sex, marital status, region, educational level, physical activity, depression
6	Ghana	Lloyd-Sherlock 2014 [18]				older age group, female sex, increasing BMI, smoker	no education, alcohol consumption, rural residence	physical activity, wealth quintile, health insurance
7	South Africa	Lloyd-Sherlock 2014 [18]				older age group, female sex, increasing BMI	tertiary educational level, alcohol consumption	smoking, residence, wealth quintile, health insurance
8	Ghana	Lloyd-Sherlock 2017 [19]				older age group (65–69 years), female sex, richer quintiles	no education, rural residence	Nil
9	South Africa	Lloyd-Sherlock 2017 [19]				older age group (75+ years), female sex, richest quintile	Higher (tertiary) educational level	urban-rural residence
10	Senegal	Macia 2012 [30]	Older age, higher BMI		Sex, educational level, marital status, doctor visits in the previous years	Older age group; overweight (BMI ≥25 kg/m^2^)		Sex, educational level, marital status
11	Kenya	Mathenge 2010 [31]	Urban residence, Kikuyu tribe			Urban residence, Kikuyu tribe		Adjusted for age, sex, SES quartile, BMI, WHR, smoking status, alcohol use, diabetes (by measurement), cholesterol
12	Ghana	Minicuci 2014 [22]	Older age, marital status, ethnicity, residence, wealth quintile		sex	older age, urban residence, overweight/obesity	underweight BMI < 18.5; Upper East and Upper West regions	sex, educational level, administrative regions except Upper East and Upper West
13	Ghana	Nuertey 2017 [34]	overweight/obesity			overweight/obesity		Model adjusted for sex, religion, region of residence, ethnicity, marital status, education, social class, use of eye glasses, diabetes, arthritis, previous surgeries, mean arterial pressure, smoking status, hearing loss, total cholesterol, triglycerides, low density lipoprotein, coronary risk ratio
14	South Africa	Peltzer 2013 [23]	Females, Older age (60–69-year group), coloured race; self-reported conditions (diabetes, stroke, arthritis), overall self-reported health status (moderate), being overweight (BMI ≥25 kg/m^2^); having had ≥5 outpatient visits in past 12 months; overweight, severe dependency	alcohol use in past month; underweight (BMI < 18.5 kg/m^2^)	marital status, educational level, wealth, urban-rural residence, physical activity, daily tobacco use, fruit and vegetable intake, social cohesion index	Coloured race, self-reported history of stroke, frequent outpatient visits in the past 12 months	alcohol use in past month	Age, sex, marital status, past medical history of diabetes, subject health status, activity limitation (dependency)
15	CAR, Congo	Pilleron 2017 [36]	Female sex, increasing age, living in Republic of Congo, urban residence, previous occupation as craftsman/storekeeper or being jobless, increasing BMI, high cholesterol, eating 3 or more meals daily	current smoker, high physical activity ≥150 min/wk	marital status, primary education, diabetes by measurement, alcohol intake	increasing age, living in Congo, previous occupation as craftsman/storekeeper or being jobless, increasing BMI, eating 3 or more meals daily	current or ex-smoker, high physical activity ≥150 min/wk	sex, rural-urban residence, primary education, cholesterol level, diabetes, alcohol consumption
16	Nigeria	Raji 2017 [37]	female sex, unmarried, urban residence, never smoked, never drank alcohol, overweight/obesity		older age, educational level, socioeconomic class, self-reported diabetes, history of transient ischaemic attack, diagnosis of lifetime depression	high educational level, urban or semi-urban residence, overweight/obesity	female sex, currently unmarried	older age, high SES, current smoking, current alcohol consumption, absence of self-reported diabetes
17	Uganda	Scholten 2011 [38]				Older age, urban residence	HIV infection (on or not on ART)	sex, marital status, education
18	Cameroon	Tianyi 2017 [39]	overweight/obesity	occupational level (≥medium)	age, sex, marital status, illiteracy, occupational level	overweight/obesity		age, sex, marital status, illiteracy, occupational level
19	Ghana	Tyrovolas 2015 [20]				higher BMI categories, self-reported diabetes, self-reported stroke, higher fruit intake	underweight BMI < 18.5	Alcohol consumption, smoking, physical activity, vegetable intake, education, wealth. Adjusted for age, sex and marital status.
20	South Africa	Tyrovolas 2015 [20]				higher BMI categories, alcohol intake, ex-smoker, low physical activity, self-reported stroke, higher vegetable intake	heavy alcohol user, low level physical activity, secondary level of education	Self-reported diabetes, fruit intake, wealth. Adjusted for age, sex and marital status.

*ART* antiretroviral therapy, *BMI* body mass index, *CAR* Central African Republic, *DHTN* diastolic hypertension, *HTN* hypertension, *NS* not statistically significant, *SAGE* Study on Global Ageing and Adult Health, *SHTN* systolic hypertension, *SES* socio-economic status, *WHR* waist-to-hip ratio

**Table 4:** Demographic and socioeconomic determinants associated with hypertension in older adults in Africa

No.	Country	Reference	Age	Sex	Residence	Education	Wealth	Occupation
1	Nigeria	Abegunde 2013 [24]		Compared with males: Females OR = 1.551; (1.01–2.39; *P* = 0.046)			Decreasing monthly income: OR = 0.798; 95%CI = 0.677–0.940; *P* = 0.007	
2	Ghana	Boateng 2017 [21]		Compared with males: Female sex RR 1.33 (1.04, 1.69) *p* ≤ 0.05 (Stage 1 SHTN); RR = 1.66 (1.28, 2.15) p ≤ 0.001 (Stage 2 SHTN).	Compared with urban residents: rural RR = 0.86 (0.65, 1.13), NS (Stage 1 SHTN); RR = 0.79 (0.59, 1.05), NS (Stage 2 SHTN).		Compared with the poorest adults: the next poor quintile group 2, RR 1.47 (1.02, 2.09) *p* ≤ 0.05 (Stage 1 SHTN); all other quintile groups NS for stage 1 or stage 2 SHTN	
3	Ghana	Boateng 2017 [21]		Compared with males: Female sex RR 0.91 (0.69, 1.19) NS (Stage 1 DHTN); RR = 0.79 (0.61, 1.01) NS (Stage 2 DHTN).	Compared with urban residents: rural RR = 0.91 (0.69, 1.19), NS (Stage 1 DHTN); RR = 0.79 (0.61, 1.02), NS (Stage 2 DHTN).		Compared with the poorest adults: the next poor quintile group 2, RR 1.47 (1.03, 2.13) p ≤ 0.05 (Stage 1 DHTN); all other quintile groups NS for stage 1 or stage 2 DHTN	
4	Tanzania	Dewhurst 2013 [25]	Compared with 70–74-year age group: ≥85y OR = 1.53 (1.13–2.08); 75–79 years OR 1.13 (0.89–1.44); 80–84 years OR 1.18 (0.88–1.58)	Compared with males: female, OR 1.80 (1.48–2.20)	Compared with lowland village dwelling: upland dwelling, OR = 1.52 (1.21–1.92)			
5	Tunisia	Hammami 2011 [26]	NS	NS	NS	NS		
6	Ghana	Lloyd-Sherlock 2014 [18]	50–54 (ref.): 55–59 years, OR 1.12 (0.91–1.38); 60–64 years, OR 1.24(0.99–1.56); 65–69 years, OR 1.53 (1.21–1.95); 70–74 years, OR 1.32 (1.04–1.67); 75+ years, OR 1.28 (1.02–1.61)	Female, OR 1.16 (1.00–1.35)	Urban (ref.): rural OR 0.73 (0.63–0.85)	Primary (ref.): None, OR 0.82 (0.69–0.98); Secondary, OR 0.76 (0.53–1.10); Higher, OR 0.96 (0.78–1.18)	Poorest wealth quintile (ref.): Q2, OR 1.11 (0.90–1.38); Q3, OR 1.77 (0.95–1.45); Q4, OR 1.77 (0.94–1.47); Richest, OR 1.16 (0.91–1.48)	
7	South Africa	Lloyd-Sherlock 2014 [18]	50–54 (ref.): 55–59 years, OR 1.31 (1.13–2.07); 60–64 years, OR 1.53 (0.99–1.56); 65–69 years, OR 1.50 (1.07–2.09); 70–74 years, OR 1.53 (1.02–2.29); 75+ years, OR 1.83 (1.25–2.68)	Female, OR 1.29 (1.05–1.59)	Urban (ref.): rural OR 1.04 (0.83–1.31)	Primary (ref.): None, OR 1.11 (0.85–1.43); Secondary, OR 0.77 (0.57–1.04); Higher, OR 0.67 (0.48–0.93)	Poorest wealth quintile (ref.): Q2, OR 0.87 (0.64–1.17); Q3, OR 1.03 (0.75–1.42); Q4, OR 1.26 (0.90–1.78); Richest, OR 1.26 (0.88–1.80)	
8	Ghana	Lloyd-Sherlock 2017 [19]	50–54 (ref.): 55–59 years, OR 1.08 (0.85–1.38); 60–64 years, OR 1.19 (0.91–1.54); 65–69 years, OR 1.36 (1.04–1.78); 70–74 years, OR 1.20 (0.92–1.58); 75+ years, OR 1.09 (0.84–1.40)	Female, OR 1.31 (1.12–1.54)	Urban (ref.): rural OR 0.64 (0.52–0.79)	Primary (ref.): None, OR 0.80 (0.66–0.98); Secondary, OR 0.77 (0.51–1.16); Higher, OR 0.94 (0.74–1.19)	Poorest wealth quintile (ref.): Q2, OR 1.20 (0.99–1.46); Q3, OR 1.36 (1.06–1.74); Q4, OR 1.55 (1.19–2.01); Richest, OR 1.68 (1.28–2.21)	
9	South Africa	Lloyd-Sherlock 2017 [19]	50–54 (ref.): 55–59 years, OR 1.36 (0.93–1.98); 60–64 years, OR 1.58 (0.99–2.54); 65–69 years, OR 1.52 (1.03–2.26); 70–74 years, OR 1.38 (0.79–2.41); 75+ years, OR 1.62 (1.09–2.40)	Female, OR 1.42 (1.09–1.85)	Urban (ref.): rural OR 1.42 (1.09–1.85)	Primary (ref.): None, OR 1.09 (0.81–1.48); Secondary, OR 0.86 (0.57–1.28); Higher, OR 0.59 (0.39–0.90)	Poorest wealth quintile (ref.): Q2, OR 1.00 (0.62–1.62); Q3, OR 1.24 (0.76–2.04); Q4, OR 1.43 (0.88–2.33); Richest, OR 1.80 (1.04–3.12)	
10	Senegal	Macia 2012 [30]	50–59 year age group (ref.): 60-69y, OR = 1.94 (1.22–3.07), *p* < 0.01; ≥70y, OR = 2.54 (1.45–4.44), p < 0.01	Males (ref.): Females OR 1.01 (0.66–1.56)		Schooling ≥9 years (ref.): None, OR 1.28 (0.73–2.23);1–8 years, OR 1.23 (0.71–2.14)		
11	Kenya	Mathenge 2010 [31]	Age adjusted	Sex adjusted	Urban (ref.): rural OR 0.77 (0.67–0.91)	SES adjusted	SES adjusted	
12	Ghana	Minicuci 2014 [22]	50–64 (ref.): 65–74 years, OR 1.28 (1.05–1.55); ≥75 years, OR 1.14 (0.90–1.45)	Males (ref.): Females OR 1.14 (0.94–1.38)	Urban (ref.): rural OR 0.77 (0.61–0.97)	None (ref.): Primary, OR 1.07 (0.89–1.30); Secondary, OR 0.90 (0.60–1.35); High school completed, OR 1.15 (0.90–1.46); Tertiary or higher, OR 0.67 (0.43–1.06)		
13	Ghana	Nuertey 2017 [34]	Age adjusted	Sex adjusted	Region of residence adjusted	Education adjusted		Social class adjusted
14	South Africa	Peltzer 2013 [23]	50–59 (ref.): 60–69 years, OR 1.30 (0.94–1.79); ≥70 years, OR 1.19 (0.80–1.78)	Males (ref.): Females OR 1.18 (0.61–1.25)				
15	CAR, Congo	Pilleron 2017 [36]	Age (continuous variable); OR = 1.02 (95% CI 1.01–1.04)	NS, OR = 0.98 (0.70–1.37); *p* = 0.924	Urban (ref.): rural OR 0.92 (0.71–1.19), *p* value = 0.528. Living in Congo vs. CAR: OR 1.68 (1.31–2.16) *p* < 0.001	Having primary education vs. not having it; OR = 1.09 (0.81–1.46); *p* = 0.566		Compared with previous occupation as employee/government employee: craftsman/storekeeper OR 1.59 (1.10–2.31); Farmer/breeder/fisherman OR = 1.65 (1.13–2.41); Jobless OR = 1.83 (1.03–3.28).
16	Nigeria	Raji 2017 [37]	Age > 69 years, OR 1.16 (0.78–1.72), p value = 0.443	Males (ref.): Females OR 0.62 (0.48–0.79), p value = 0.001; NS	Urban/semi-urban (ref.): Rural OR 0.53 (0.72–0.98); *p* = 0.04	Non-high education (ref.): High educational level OR 2.55 (1.02–6.38), p value = 0.045		High SES, OR 1.50 (0.80–2.86), NS
17	Uganda	Scholten 2011 [38]	50–59 (ref.): 60–69 years, OR 1.13, NS; ≥70 years, OR 2.48; *p* = 0.01	Males (ref.): Females OR 1.47; NS	Urban (ref.): Rural OR 0.57; p = 0.04	Primary (ref.): None, OR 1.35, NS; Secondary or higher, OR 1.38; NS		
18	Cameroon	Tianyi 2017 [39]	NS	NS		NS		Compared with low occupational level (unskilled workers): medium or high level occupational level: OR = 0.56 (0.23–1.32); *P* = 0.183
19	Ghana	Tyrovolas 2015 [20]	Adjusted for age, sex and marital status	Adjusted for age, sex and marital status		Compared with ≤primary level: Secondary OR 1.11 (0.89–1.38), NS; ≥Tertiary, OR 0.66 (0.41–1.06), NS	Compared with middle wealth quintile: Poorest OR 0.83 (0.65–1.07), NS; Poorer, OR 1.02 (0.80–1.30); Richer, OR 1.10 (0.86–1.41); Richest, OR 1.15 (0.87–1.52)	
20	South Africa	Tyrovolas 2015 [20]	Adjusted for age, sex and marital status	Adjusted for age, sex and marital status		Compared with ≤primary level: Secondary OR 0.66 (0.46–0.95), p < 0.05; ≥Tertiary, OR 0.66 (0.41–1.06), NS	Compared with middle wealth quintile: Poorest OR 0.85 (0.54–1.35), NS; Poorer, OR 0.78 (0.49–1.24); Richer, OR 0.87 (0.54–1.39); Richest, OR 1.14 (0.69–1.89)	

NS = not statistically significant; OR = odds ratio; SES = socioeconomic status

In multivariate analyses, older age group and female sex were often independently associated with hypertension. Of the 16 studies providing 20 data contributions with multivariate analysis, age was either not included in the logit models in two studies (three data points) [21, 24] or was adjusted for without being assigned an effect size in three studies (four data points) [20, 31, 34] (Table 4). In the remaining 11 studies (13 data contributions), older age group predicted hypertension in a total of nine models from seven studies [18, 19, 22, 25, 30, 36, 38] but was not statistically significant in four studies (four data contributions) [23, 26, 37, 39] (Tables 3 and 4). The highest odds ratio was observed in a study in Senegal in which the odds of being hypertensive among subjects aged 60–69 years and those aged ≥70 years was respectively 1.9 and 2.5 times that in those aged 50–59 years [30] (Table 4). In other studies, however, the relationship between older and the younger age groups was erratic and non-monotonic. For example, in Ghana, the odds ratio associated with hypertension in those aged 65–69 years (compared with those aged 50–54 years) was higher than that of the 70–74 or ≥ 75-year age groups [18].

Female sex was a frequent predictor of hypertension in multivariate analyses. The adjusted odds of hypertension were up to 80% higher in females than in males [18, 19, 21, 24, 25] (Table 3). However, female sex was associated with lower adjusted odds of hypertension in one study in Nigeria [37]. It was not independently associated with hypertension in the multivariate analyses [22, 38], having lost its statistically significant relationship observed in the bivariate analysis in three studies [23, 26, 36] (Table 2). Similarly, the statistically significant relationship between older age and hypertension disappeared in the multivariate analyses in two of these studies [23, 26].

***Residence*** In studies in which the residential distribution of the prevalence of hypertension was reported, it was higher in urban than in rural areas by up to 15 percentage points [22, 24, 31, 36, 43]. In one study in Nigeria, the prevalence increased along a gradient from rural, semi-urban to urban areas [37]. However, in South Africa, the prevalence was slightly higher in rural (77.5%) than in urban populations (77.2%) [23]. The urban-rural difference was statistically significant in studies in Ghana [22], Kenya [31] and Uganda [38] but not in studies in Tunisia [26] or South Africa [23] (Tables 3 and 4). In multivariate analyses, urban residence was often significantly associated with hypertension, as in Ghana, Nigeria and Kenya with adjusted odds ratios about 30–40% higher than in the rural areas [22, 31, 37, 38]. It was not significantly associated with hypertension in other studies in Ghana and CAR/Congo [21, 36]. In the multi-country SAGE study in older adults, urban residence predicted hypertension in Ghana in two different models but not in South Africa [18, 19] (Table 4).

The adjusted odds ratio associated with hypertension among elderly subjects living in Congo was 68% higher than that of those living in the CAR [36]. In Tanzania, hypertension in elderly aged ≥70 years was more frequently associated with residence in upland than in lowland villages [25]. Compared with those residing in the Greater Accra Region, older adults in two of the poorest regions in Ghana, the Upper East and Upper West regions had 63–76% lower odds of having hypertension after controlling for other factors [22]. Otherwise, residence in the other administrative regions of Ghana was not independently associated with hypertension.

***Other demographic factors*** Marital status did not make a statistically significant difference to the prevalence of hypertension in bivariate analyses [26, 30, 36, 39]. For example, in Senegal, 66.1% of married subjects compared with 63.3% of unmarried subjects had hypertension [30]. In multivariate analyses, marital status was not independently associated with hypertension in the studies in which this relationship was assessed [23, 26, 30, 38, 39] except in Nigeria where being unmarried had a protective effect [37] (Table 4).

Only two studies evaluated ethnicity and found that it was independently associated with hypertension in the rural Hai district of Tanzania [25] and in the Nakuru district of Kenya [31] (Tables 3 and 4). The adjusted odds ratios associated with hypertension comparing the Chagga tribe to the non-Chagga tribes and comparing Kikuyus to the Kalenjins were 1.65 (95% CI 1.18–2.30) [25] and 1.4 (95% CI 1.2–1.7) [31] in the two respective studies (Table 4). The odds of having hypertension among coloured South African older adults was 89% greater than that among black Africans [23]. Practitioners of traditional religion faith in Ghana had 0.35–0.38 times the adjusted odds of having stage 2 SHTN or DHTN as those with no religion [21].

##### Socioeconomic factors

***Occupation and educational level.*** In bivariate analyses, previous occupation as craftsman, storekeeper or being jobless was associated with being hypertensive in the CAR and Congo [36] (Table 3). In Cameroon, having medium or high-level occupation (higher than unskilled work) protected against having hypertension [39]. Otherwise, socioeconomic factors such as educational level, wealth or occupational level were not significantly associated with hypertension in Senegal, South Africa, CAR, Congo or Cameroon [23, 36, 39].

In South Africa, 76.4% of older adults with no schooling compared with 75.8% of those with ≥12 years of schooling had hypertension [42]. The prevalence of hypertension was higher among the groups with intervening schooling year duration - 78.9% among those with less than 7 years and 79.2% among those with 8–11 years of schooling. In Tunisia, the prevalence of hypertension among elderly subjects aged years declined with increasing educational level but the difference was not statistically significant [26]. It was 53.9% among illiterate subjects, 47.1% among those with primary education and 31.3% in those with secondary or higher-level education.

The association between previous employment as a craftsman, storekeeper, farmer, breeder, fisherman or being jobless and hypertension in the Central African Republic and Congo [36] persisted in the multivariate analysis (Table 3). Similarly, the absence of a statistically significant association between educational level and hypertension in Central African Republic, Congo, Nigeria and Senegal remained after adjusting for other variables [30, 36, 37]. The results were similar in Ghana [22] and Uganda [38] where educational level did not correlate with hypertension.

Multivariate analyses of the multi-country SAGE dataset gave discordant results (Tables 3 and 4). Compared to the primary level, higher (tertiary) level educational attainment in South Africa predicted lower prevalence of hypertension whereas, in Ghana, it was no education that did so [18, 19]. Contrary to these findings, another group evaluating different variables on the same multi-country SAGE dataset found that, compared to primary or lower level educational attainment, secondary education level was protective of hypertension in South Africa [20]. This group reported that neither secondary nor tertiary level educational attainment was significantly associated with hypertension in Ghana.

***Health insurance and wealth quintile*** Only one study, a SAGE multi-country study, assessed the association between having health insurance and being hypertensive in a multivariate model [18]. It found that having health insurance was not significantly associated with hypertension in Ghana or South Africa.

**Fig. 2:**
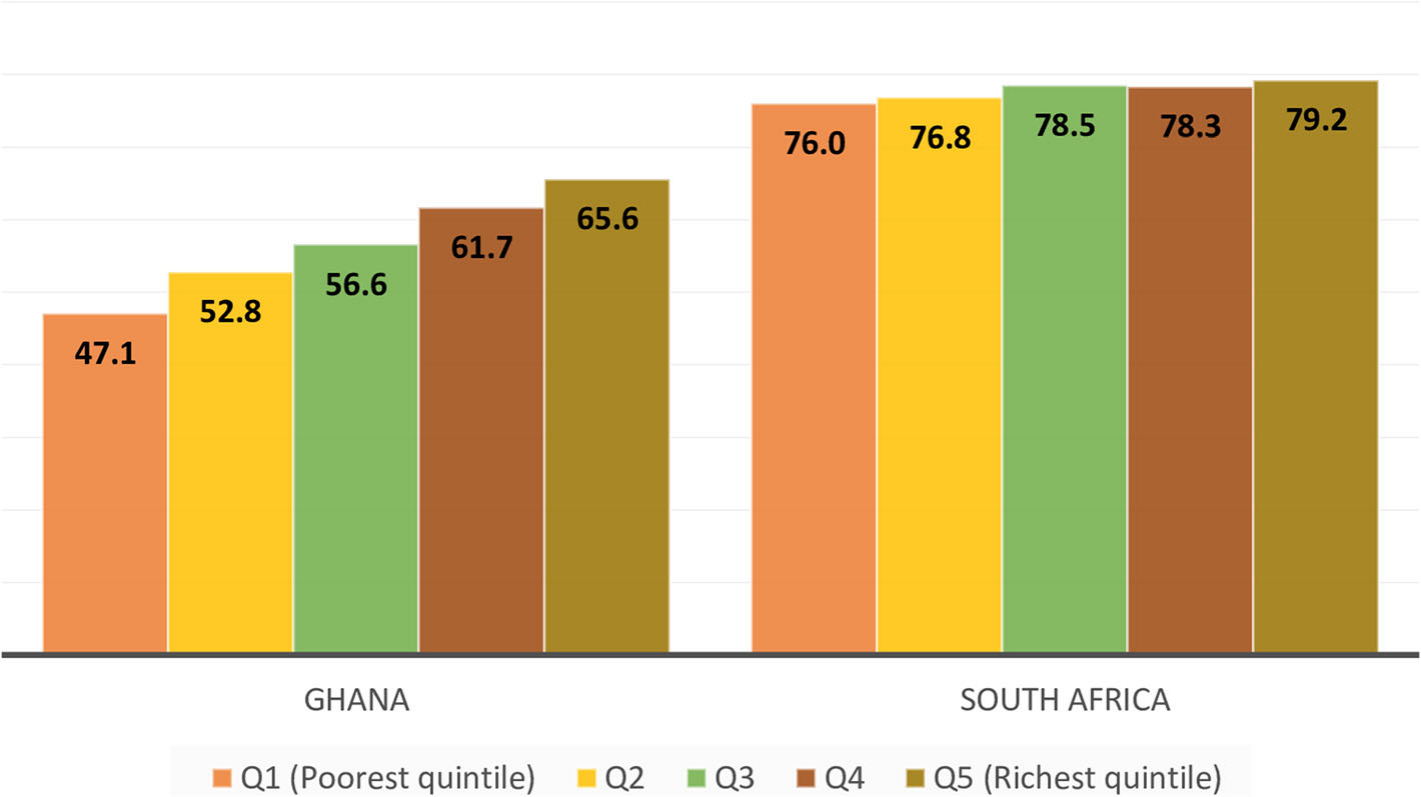
Prevalence of hypertension by wealth quintile. Source: Lloyd-Sherlock et al. 2017 [19]

There were contrasting patterns in the relationship between wealth quintile and the prevalence of hypertension in Ghana and South Africa (Fig. 2). In Ghana, the prevalence increased with increasing wealth quintile whereas in South Africa, the difference between the different quintiles groups was minimal [19]. The ratio of the prevalence in the richest and poorest quintiles was 1.39 and 1.04 in the two respective countries.

Wealthier quintile groups were independently associated with hypertension in older adults in the SAGE study in Ghana [19, 21] (Tables 3 and 4). Consistent with the positive gradient economic observed in the crude analysis in Ghana, the adjusted odds ratio associated with hypertension in the multivariate analysis increased steadily from 1.20 in the second wealth quintile (Q2), to 1.36 in Q3, 1.55 in Q4 and 1.68 in the richest quintile group when compared to the poorest wealth quintile [19]. However, no such dose-response gradient was observed among the subjects in South Africa in whom only the adjusted odds ratio comparing the richest to the poorest wealth quintile was statistically significant. In two other multi-country publications of the same SAGE data on Ghana and South Africa which fitted models with additional variables, wealth quintiles were not associated with hypertension [18, 20]. Neither were they associated with stage 1 or stage 2 systolic hypertension (SHTN) or diastolic hypertension (DHTN) in Ghana [21]. Consistent with the SAGE Ghana pattern, another multivariate analysis concluded that low monthly income of adults aged ≥60 years in Nigeria protected against hypertension.

##### Lifestyle factors

***Body mass index.*** In both bivariate and multivariate analyses, overweight as well as general and abdominal obesity was consistently associated with a higher prevalence of hypertension [26, 36]. Overweight/obese (BMI ≥25 kg/m^2^) subjects were 1.2 to 2.0 times as likely as non-overweight subjects (BMI < 25 kg/m^2^) to have hypertension, with the difference being statistically significant [26, 30]. The relationship followed a dose-response pattern in Tunisia with prevalence at 29.9% among normal weight elderly subjects (BMI 18.0–24.9 kg/m^2^), 49.1% among those overweight non-obese (BMI 25.0–29.9 kg/m^2^) and 64.2% among those obese (BMI ≥30.0 kg/m^2^) [26]. Conversely, being underweight (BMI < 18.5 kg/m^2^) was associated with a lower prevalence of hypertension in South Africa [23].

In all the models in which it was evaluated whether as a categorical or continuous variable, BMI or overweight/obese was strongly, consistently and independently correlated with hypertension [18–21, 23–26, 30, 34, 36, 39] (Table 5). The adjusted odds of being overweight or obese in subjects with hypertension was up to 3.7 times that in non-overweight/non-obese subjects [37, 39]. It increased with increasing BMI status [20, 26, 36]. Compared with those with normal BMI, the adjusted odds ratio associating underweight adults involved in the cross-country SAGE study with hypertension (compared with adults with normal BMI) was protective in Ghana but not in South Africa [18, 20].

**Table 5:** Comorbidities and other determinants associated with hypertension in older adults in Africa

No.	Country	Reference	Physical activity	Obesity or adiposity	Alcohol	Diabetes	Other determinants
1	Nigeria	Abegunde 2013 [24]		Compared with normal BMI: obese OR = 2.8 (1.52–5.29); *p* = 0.001			
2	Ghana	Boateng 2017 [21]		Compared with BMI normal weight category: underweight RR = 0.65 (0.40, 0.81) *p* ≤ 0.01 (Stage 1 SHTN); RR = 0.57 (0.41, 0.81), *p* ≤ 0.001 (Stage 2 SHTN). Overweight RR = 1.73 (1.24, 2.40), p ≤ 0.001 (Stage 2 SHTN) Obese RR = 1.64 (1.07, 2.51) *p* ≤ 0.05 (Stage 1 SHTN); RR = 1.81 (1.19, 2.77), p ≤ 0.01 (Stage 2 SHTN)	Consumes alcohol compared with does not consume alcohol: RR 1.41 (1.08, 1.85) p ≤ 0.05 (Stage 1 SHTN); RR = 0.89 (0.68, 1.18) NS (Stage 2 SHTN).		Compared with subjects with no religion: Traditional religion, RR 0.38 (0.19, 0.75) p ≤ 0.01 (Stage 2 SHTN)
3	Ghana	Boateng 2017 [21]		Compared with BMI normal weight category: underweight RR = 0.75 (0.54, 1.02) NS (Stage 1 DHTN); RR = 0.63 (0.47,0.85), NS (Stage 2 DHTN). Overweight RR = 1.51 (1.06, 2.15), p ≤ 0.05 (Stage 1 DHTN); 1.67 (1.21, 2.31), p ≤ 0.001 (Stage 2 DHTN) Obese RR = 1.79 (1.16, 2.77) p ≤ 0.01 (Stage 1 DHTN); RR = 1.77 (1.18, 2.63), p ≤ 0.01 (Stage 2 DHTN)	Consumes alcohol compared with does not consume alcohol: RR 0.94 (0.71, 1.23), NS (Stage 1 DHTN); RR = 0.91 (0.71, 1.16), NS (Stage 2 DHTN).		Compared with subjects with no religion: Other religion, RR 0.30 (0.11, 0.82) p ≤ 0.01 (Stage 1 DHTN); Traditional religion RR = 0.35 (0.19, 0.75) p ≤ 0.001 (Stage 2 DHTN)
4	Tanzania	Dewhurst 2013 [25]		Increasing BMI: OR 1.09 (1.06–1.12)			Compared with subjects of non-Chagga tribal origin: Chagga tribe, OR 1.65 (1.18–2.30)
5	Tunisia	Hammami 2011 [26]		BMI (continuous) OR 2.01 (1.5–2.5); *P* < 0.01		Self-reported diabetes OR 2.06 (1.45–3.5), *p* < 0.001	Disability OR 1.6 (0.9–2.7), p < 0.001
6	Ghana	Lloyd-Sherlock 2014 [18]	High physical activity (ref.): Moderate, OR = 1.02 (0.83–1.25); Low, OR 1.09 (0.92–1.30)	Normal BMI (ref): Underweight, OR 0.65 (0.54–0.79); Overweight, OR 1.77 (1.48–2.12); Obese, OR 2.30 (1.76–3.00)	Life-time abstainers (ref.): non-heavy drinker, OR 0.80 (0.69–0.94); Infrequent heavy drinkers, OR 1.24 (0.66–2.33); Frequent heavy drinkers, OR 1.24 (0.66–2.33)		Never smoker (ref.): Less than daily / ever smoker, OR 1.24 (1.02–1.49); Daily, OR 0.83 (0.61–1.12). Has health insurance vs. Uninsured, OR 1.08 (0.94–1.25)
7	South Africa	Lloyd-Sherlock 2014 [18]	High physical activity (ref.): Moderate, OR = 0.91 (0.66–1.26); Low, OR 0.77 (0.61–0.97)	Normal BMI (ref): Underweight, OR 0.96 (0.58–1.59); Overweight, OR 1.57 (1.29–2.06); Obese, OR 1.85 (1.43–2.39)	Life-time abstainers (ref.): non-heavy drinker, OR 0.66 (0.47–0.91); Infrequent heavy drinkers, OR 0.41 (0.25–0.67); Frequent heavy drinkers, OR 0.45 (0.17–1.15)		Never smoker (ref.): Less than daily / ever smoker, OR 1.05 (0.78–1.42); Daily, OR 1.07 (0.81–1.42). Has health insurance vs. Uninsured, OR 0.92 (0.71–1.20)
8	Senegal	Macia 2012 [30]		Compared with BMI < 25: BMI ≥25, OR = 1.86 (1.24–2.79), *p* < 0.01			Married (ref.): Not married, OR 0.81 (0.51–1.28)
9	Kenya	Mathenge 2010 [31]		BMI and WHR adjusted	Alcohol use adjusted	Diabetes (laboratory-confirmed) adjusted	Ethnicity Kalenjins (ref.): Kikuyus OR 1.4 (1.2–1.7)
10	Ghana	Minicuci 2014 [22]		Normal weight (ref.): Overweight BM 25.0–29.9 kg/m2 OR = 1.72 (1.40–2.11); Obese BMI ≥30.0 kg/m2 OR 2.03 (1.53–2.71) Underweight (BMI < 18.5 kg/m2) OR 0.63 (0.50–0.80)			Administrative region. Greater Accra (ref.): Ashanti OR 1.14 (0.78–1.66); Brong Ahafo, OR 0.98 (0.66–1.45); Central, OR 1.03 (0.69–1.52); Eastern, OR 1.03 (0.73–1.45); Northern, OR 0.90 (0.54–1.49); Upper East, OR 0.37 (0.24–0.58); Upper West, OR 0.24 (0.12–0.46); Volta, OR 0.99 (0.65–1.48); Western, OR 0.81 (0.54–1.21)
11	Ghana	Nuertey 2017 [34]		Adjusted OR 1.8 (1.5–2.0) compares overweight/obese in hypertensives and non-hypertensives		Adjusted for self-reported diabetes	
12	South Africa	Peltzer 2013 [23]		Normal weight (ref.): Overweight BMI ≥25 kg/m2 OR = 1.52 (1.15–2.01), p < 0.01 Underweight (BMI < 18.5 kg/m2) OR 0.77 (0.36–1.64	Alcohol use over past one month, OR = 0.64 (0.49–0.84)	Self-reported diabetes, OR 1.30 (0.86–1.98)	Race black African (ref.): white OR 1.23 (0.66–2.30); Coloured OR 1.89 (1.04–3.44); Indian or Asian OR 0.82 (0.47–1.42) Self-reported stroke OR 4.48 (1.48–13.59), p < 0.01 Self-reported arthritis OR 1.13 (0.75–1.69) Outpatient visits in past 12 months; Nil (ref.):1–4 visits OR 1.14 (0.80–1.63); ≥ 5 visits vs. nil, OR = 1.93 (1.48–2.51)
13	CAR, Congo	Pilleron 2017 [36]	high physical activity ≥150 min/wk. vs. < 150 min/wk.; OR = 0.75 (0.59–0.96); *p* = 0.023	BMI (continuous variable); OR = 1.09 (1.06–1.12); p < 0.001	Compared with abstainers: light OR = 0.99 (0.77–1.27); moderate to heavy OR = 0.89 (0.42–1.89); *p* = 0.953	Diagnosed diabetes OR 0.85 (0.56–1.28), *p* = 0.408	Compared with tobacco nonuser: ex-user OR = 0.65 (0.41–1.03); current smoker OR = 0.60 (0.38–0.95); other mode of intake OR = 0.81 (0.60–1.11) High cholesterol vs normal level: OR 1.33 (0.90–1.97)
14	Nigeria	Raji 2017 [37]		Overweight/obesity BMI ≥25 kg/m2 OR = 3.72 (1.47–9.40), *p* = 0.007	Never drank alcohol (ref.): Drank alcohol OR 0.84 (0.66–1.09), NS	No history of self-reported diabetes (ref.): Self-reported diabetes OR 0.91 (0.50–1.75), *p* value = 0.389	Cigarette smoking OR 0.97 (0.73–1.29), p value = 0.819
15	Uganda	Scholten 2011 [38]					Married (ref.): Not married, OR 0.92; NS HIV infection and treatment, No HIV (ref.): HIV on ART, OR 0.50, NS; HIV no ART, OR 0.23, p value = 0.01
16	Cameroon	Tianyi 2017 [39]		Overweight/obesity vs. Others, OR = 3.46 (2.38–5.03), p < 0.001			
17	Ghana	Tyrovolas 2015 [20]	Compared with high level physical activity: Moderate, OR 0.96 (0.72–1.28), NS; Low, OR 1.12 (0.91–1.38), NS	Compared with normal BMI: underweight OR 0.70 (0.55–0.90), p < 0.01; overweight OR 1.90 (1.54–2.35), p < 0.001; BMI 30.0–34.9 category OR 2.14 (1.51–3.04), p < 0.001; BMI ≥ 35 OR 2.78 (1.65–4.69), p < 0.001	Compared with never: Non-heavy OR 1.01 (0.86–1.19), NS; Heavy OR 1.07 (0.62–1.84), NS	Self-reported Yes vs No: OR 2.37 (1.51–3.74), p < 0.001	History of stroke, Yes vs. No: OR 3.45 (1.70–7.01), *p* < 0.001. Fruit servings/day, 0–1 (ref): 2–4 servings OR 1.05 (0.86–1.28); ≥5 servings, OR 1.64 (1.13–2.39), p < 0.01 Vegetable servings/day, 0–1 (ref): 2–4 servings OR 1.04 (0.84–1.27); ≥5 servings, OR 0.68 (0.35–1.34) Compared with never smoker: Current smoker OR 0.78 (0.60–1.01); Ex-smoker, OR 1.22 (0.96–1.54)
18	South Africa	Tyrovolas 2015 [20]	Compared with high level physical activity: Moderate, OR 0.76 (0.50–1.17), NS; Low, OR 0.67 (0.47–0.96), *p* < 0.05	Compared with normal BMI: underweight OR 0.84 (0.47–1.51); overweight OR 1.53 (1.07–2.19), p < 0.05; BMI 30.0–34.9, OR 1.65 (1.06–2.58), p < 0.05; BMI ≥ 35 OR 1.60 (1.04–2.44), p < 0.05	Compared with never: Non-heavy OR 0.92 (0.62–1.35), NS; Heavy OR 0.43 (0.23–0.81), p < 0.01	Self-reported Yes vs No: OR 1.61 (0.96–2.70)	History of stroke, Yes vs. No: OR 3.18 (1.36–7.43), p < 0.01 Fruit servings/day, 0–1 (ref): 2–4 servings OR 0.90 (0.65–1.24); ≥5 servings, OR 1.87 (0.63–5.54) Vegetable servings/day, 0–1 (ref): 2–4 servings OR 1.55 (1.11–2.18), p < 0.05; ≥5 servings, OR 1.20 (0.58–2.46) Compared with never smoker: Current smoker OR 1.25 (0.87–1.78); Ex-smoker, OR 1.70 (1.01–2.85), p < 0.05

ART = antiretroviral therapy; BMI = body mass index; DHTN = diastolic hypertension; HIV = human immunodeficiency virus; NS = not statistically significant; OR = odds ratio; RR = relative risk; SHTN = systolic hypertension

***Other lifestyle factors*** The relationships between other lifestyle factors and hypertension were not as remarkable as that with the BMI. In isolated studies, alcohol use in the past one month in South Africa [23] and being an former or current smoker in CAR/Congo [36] protected against having hypertension. Daily tobacco use or inadequate fruit and vegetable intake was not significantly associated with hypertension in South Africa [23]. There was an inverse association between the level of physical activity and hypertension in CAR/Congo [36]. However, in South Africa, the association was not statistically significant [23]. Frequent outpatient visits during the past 12 months was significantly associated with hypertension in South Africa [23] but not in Senegal [30]. A social cohesion index constructed to assess the extent of social engagement with society, club, union, or any organizational meeting in one study was not significantly associated with hypertension [23].

The inverse association between alcohol intake and hypertension in South Africa persisted in multivariate analysis [23]. The adjusted odds of being heavy drinker in hypertensives was 57% lower than that of being a lifetime abstainer [20]. In contrast, alcohol intake was associated with a 41% greater prevalence of stage 1 SHTN in Ghana [21]. Overall, it was not significantly associated with stage 1 or 2 DHTN or any hypertension in Ghana [20, 21], Congo or Central African Republic [36] or Nigeria [37].

The association between smoking and hypertension was inconsistent between consumption habits or countries. Current non-daily or former smoking was independently associated with hypertension as a harmful predictor in the same multi-country analyses of the SAGE study in Ghana but not in South Africa [18]. In similar analyses, former smoking was independently associated with higher prevalence of hypertension in South Africa but not in Ghana [20]. Contrary to these findings, in CAR/Congo, current smoking was associated with a lower prevalence of hypertension [36] while in Nigeria, having ever smoked tobacco was not independently associated with hypertension [37].

Concerning dietary factors, participants who ate three or more meals daily had 85% higher adjusted odds of having hypertension than those who ate one meal daily [36]. From the multi-country SAGE data analyses, higher fruit intake of ≥5 daily servings was independently associated with hypertension in Ghana but not in South Africa [20] (Table 5). Conversely, 2–4 daily vegetable servings was independently associated with hypertension in South Africa but not in Ghana [20]. In a separate model, examined and self-reported hypertension was not associated with insufficient fruit and vegetable intake in South Africa [44].

3w?>As with alcohol intake and current or former smoking, the relationship between physical activity and hypertension was erratic and somewhat contradictory. High level of physical activity was associated with a lower prevalence of hypertension in Congo and CAR [36] and a higher prevalence of hypertension in South Africa [18, 20] (Tables 4 and 5). In Ghana and Tunisia, physical activity was not significantly associated with hypertension [18, 20, 26].

The positive relationship observed between frequent outpatient visits in the preceding 12 months and hypertension in South Africa remained in the multivariate analysis with adjusted odds ratio 1.93 (95% CI 1.48–2.51) (Table 5) [23].

##### Comorbidity

Having a history of self-reported diabetes, stroke or arthritis was associated with hypertension in Tunisia or South Africa [23, 26] but not in Nigeria [37]. Similarly, dependency, disability or activity limitation was associated with hypertension [23, 26]. The presence of laboratory-confirmed diabetes was, however, not associated with hypertension in CAR/Congo [36]. A subjective self-appraisal of being moderately well was associated with hypertension in South Africa but not having bad or very bad health [23]. A diagnosis of lifetime depression was not associated with hypertension in elderly subjects in Nigeria [37].

In the multivariate analyses, a medical history of diabetes and a subjective status of moderate health among older adults in South Africa lost their statistically significant association with hypertension [23]. Self-reported diabetes remained significantly associated with hypertension in Tunisia [26] and Ghana [20] (Tables 4 and 5). As in the crude analyses, it was not independently associated with hypertension in CAR/Congo [36], Nigeria [37] or South Africa [20, 23]. On the other hand, history of a stroke was a very strong predictor of hypertension in both Ghana and South Africa with adjusted odds ratios of 3.45 and 3.18 respectively [20].

High cholesterol among elderly subjects in Congo and CAR [36] was associated with hypertension in the crude analysis but this association disappeared in the adjusted analysis (Table 3). In the multivariate analyses, dependency, activity limitation or disability was significantly associated with hypertension in Tunisia [26] but not in South Africa [23] or Tanzania [25]. Hypertension and self-reported arthritis were not associated with each other both in the crude and adjusted analyses in South Africa [23, 45].

Compared with those who were not infected, older adults in Uganda who infected with the human immunodeficiency virus (HIV) and were on antiretroviral therapy had 77% lower odds of having hypertension after adjusting for sociodemographic variables [38].

##### Summary of determinants

From the bivariate analyses, the positive risk factors frequently associated with hypertension in older adults included older age group, female sex, urban residence, ethnicity, self-reported diabetes or stroke, and overweight/obesity. There were no consistent protective factors. The relationship between socioeconomic factors such as occupation, education and wealth quintile with hypertension was variable as was doctor visits in the past year. Smoking and alcohol intake did not emerge as harmful risk factors for hypertension. Marital status and religion were of no import to the presence of hypertension in older adults.

The strongest and most robust predictors of hypertension were overweight/obesity or increasing BMI and a history of stroke. Older age and female sex were frequent predictors in the multivariate analyses. Marital status, educational attainment and not having a health insurance were mostly not associated with hypertension. The association between lifestyle variables (such as alcohol intake, current or former smoking, fruit and vegetable intake and physical activity) and most comorbid factors with hypertension was inconsistent.

## Discussion

To our knowledge, this is the first reported comprehensive systematic review of the literature on the determinants of hypertension in older adults in Africa. The major strengths of this review are the inclusion of a wide scope of studies published in multiple languages and having varied study designs. We assessed the relationship between a wide range of demographic, socioeconomic, lifestyle and comorbid covariates with hypertension from both bivariate and multivariate analyses. Most of the studies were deemed to have low or moderate risk of bias. The review highlights the dearth of studies on determinants of hypertension in this age group in Africa with coverage of only twelve African countries.

Consistent with other reviews in Africa, our review showed that older age group and overweight/obesity were independently associated with hypertension [7, 11, 46, 47]. These same variables are also positive predictors of diabetes among older adults in Africa [48]. Our findings contrast with those of a recent meta-analysis of hypertension in older people in Africa which did not find any significant difference in the prevalence across age groups [49]. There is a biological basis for the increase in hypertension with age that is related to changes in the arterial structure and function, notably arterial stiffening with adverse consequences on cardiac structure and function [50, 51]. There is also a decline in plasma renin activity, impaired renal function and homeostatic mechanisms associated with an ageing kidney. It is ironic that the older age group which suffers the highest relative burden of hypertension is often neglected in the public health agenda in Africa [52, 53]. This situation is unacceptable considering the established evidence that cardiovascular diseases in old age can be prevented or well managed to promote a good quality of life [50, 54, 55].

As in the current review, we found in a previous systematic review that obesity or adiposity measures were strong predictors of hypertension among different cadres of workers in West Africa [11] with, for example, adjusted odds ratio of about 3.4 among health workers in a teaching hospital in Nigeria [56]. In a recent analysis of demographic and health survey results in five sub-Saharan countries, the adjusted odds ratios for hypertension was 2.44 (95% CI 2.19–2.72) and 5.34 (95% CI 4.75–5.99) among overweight and obese women respectively compared with women of normal BMI [57].

A meta-analysis of prospective studies published up to January 2017 estimated that the risk of hypertension increased by 49% for every five-unit increment in BMI, by 27% per 10 cm increase in waist circumference, and by 37 and 74% per 0.1-unit increment in waist-to-hip ratio and waist-to-height ratio respectively [58]. Another meta-analysis of published articles up to January 2016 estimated that losing excess weight may reduce the risk of hypertension by 24 to 40% in people who are overweight and by 40 to 54% in people who are obese [59]. As with older age, the close epidemiological and possibly causal link between obesity and hypertension is biologically plausible [60].

Urbanization appears to provide the catalyst for the obesity epidemic in Africa. In West Africa, a meta-analysis revealed that the prevalence of obesity increased by 114% over a 15-year period from 1990 to 2014 whereas the much lower prevalence remained stable in the rural populations [61]. There has been a rapid growth of the fast food industry in urban areas as incomes of the middle class along with increased opportunities and preferences for eating outside of home [62]. It is likely that the obesogenic environment engendered by urbanization, trade liberalization, growing food and beverage food industry and changing lifestyles contribute to the development and progression of obesity from younger ages [63]. Fortunately, there is now growing awareness of healthy food practices although these practices may be influenced by individual finances, physical, psychological and community factors [64].

The sex-differences in the prevalence of hypertension in Africa have been inconsistent. Some reviews have found minimal non-statistically significant differences [7, 46, 47, 49] while others have found greater prevalence among men [65, 66]. Unlike our previous review in which we identified male sex as one of the determinants of hypertension among workers in West Africa [11], we found in the current review that female sex was independently associated with hypertension in most studies among older adults in Africa. A global review found a higher mean blood pressure and age-standardized prevalence of hypertension among men [67]. The pattern of lower prevalence of hypertension in premenopausal women than men and the subsequent higher prevalence in postmenopausal women may be typical [68, 69]. Multiple mechanisms such as androgen-mediated increase in angiotensinogen leading to increase in endothelin-mediated vasoconstriction; oxidative stress; sympathetic nervous system activation; and increased anxiety or depression have been proposed as possible explanation for the higher prevalence in postmenopausal women [69]. Besides their higher odds for hypertension, older women are also at a significantly higher risk of having multiple risk factors for NCDs [42].

In the bivariate analysis, the prevalence of hypertension in older adults was almost always higher in urban than in rural populations in our review. In multivariate analysis, however, the greater odds of hypertension associated with urban residence was less consistent. Other systematic reviews in Africa have made similar observations [46, 47, 65, 70]. It appears the gap in the prevalence between urban and rural areas has been narrowing [71–73]. In a recent study in Zambia, the prevalence in rural populations was twice that in urban population [74].

Except in Nigeria where not being married was protective, marital status was not independently associated with hypertension. The reason for the protective effect of unmarried status of older adults for hypertension in Nigeria is not clear from the primary study included in the review. In a meta-analysis involving studies from western countries, being married was independently associated with a greater adjusted odds of having nocturnal dipping and lower mean night-time SBPs among subjects participating in a controlled dietary intervention [75]. This may be because married people have a better satisfaction with life [76] or may be due to social-cognitive factors, neuroendocrine processes, biological mediators and health behaviours [75].

Among the socioeconomic factors, we found that the association between education and wealth index with hypertension in older adults in Africa was inconsistent. We observed similar inconsistent results with education in the systematic review of hypertension among workers in West Africa [11]. The direction of the association between education and hypertension among older adults in the two SAGE countries, Ghana and South Africa was discordant [18]. In the analysis involving all the six low- and middle-income SAGE countries (China, Ghana, India, Mexico, Russia, and South Africa), education was not independently associated with hypertension in participants aged ≥18 years [77]. The prevalence of hypertension has been shown to increase with increasing illiteracy in some reviews [47] and multi-country analyses [57]. Since in low-income countries, those with higher education tend to be overweight or obese [78, 79], it may have been expected that higher education would predict hypertension if the relationships were linear or unconfounded. Longitudinal studies could help define how the relationship between education and obesity differ over the life course [78].

As with education, we observed a somewhat discordant relationship between wealth and hypertension among older adults in the SAGE studies in Ghana and South Africa [19]. In South Africa, as in Russia, with high average prevalence of hypertension, the socioeconomic gradient by wealth quintiles was almost flat [19]. In contrast, in Ghana and India, the socioeconomic gradient relating wealth quintile with hypertension in older adults was positive with the ratio of the prevalence between the richest and poorest wealth quintile groups being in the order of 1.4–1.6. In the two other SAGE countries, China and Mexico, which had a similar average prevalence of hypertension, there was less clear pattern and the socioeconomic gradient appeared negative.

As we found for older adults involved in SAGE Ghana [19], a recent analysis of studies among women aged 15–49 years in five sub-Saharan African countries also found that increasing wealth index was independently associated with hypertension along a dose-response gradient [57]. Clearly, the relationship between wealth and hypertension is context-specific. Further studies are required to more clearly define the socioeconomic predictors of hypertension and its complications, particularly in poor households who tend to be less aware of their disease and achieving worse outcomes [19].

With regards to lifestyle factors, we found an inconsistent relationship between physical activity and alcohol intake with hypertension in the current review. The cross-country analysis of the SAGE data of participants aged ≥18 years in the six SAGE countries did not find any independent association between exercise and hypertension [77]. Nevertheless, the role of physical activity in improving cardiovascular outcomes in older adults is well-recognized [80–82]. The inconsistent findings may be related to methodological issues such as the study design, study population and sample size.

As with physical activity, we found an inconsistent relationship between alcohol intake and hypertension in older adults in our review. This contrasts with the finding in our previous review among workers in West Africa in which we found that alcohol intake was generally associated with hypertension [11]. The protective effect of moderate drinking against cardiovascular disease has been widely publicized for many years [83, 84]. Recent evidence is emerging from cohort studies that alcohol at any level increases the risk of incident hypertension [85]. A meta-analysis found that, compared with abstainers, light drinking (1 to 2 drinks/day) in men increased the risk of hypertension by 19%, with the risk increasing in a dose-response manner to 74% in those drinking ≥5 drinks daily [85]. In women, the increased risk was observed in those taking two or more standard drinks daily. No level of alcohol consumption was protective in men or women. Consistent with this finding, a meta-analysis found that a reduction in alcohol intake in people who drank more than two drinks per day in the UK was associated with increased blood pressure reduction [86]. Existing recommendations to reduce alcohol intake among older adults and to discourage middle-aged adults from taking up regular alcohol intake may, therefore, be pertinent [50, 87, 88]. In the current review, the inverse association of alcohol intake and hypertension in South Africa among older adults is inconsistent with recent findings and may be due to methodological differences in study design, study population, exposure to alcohol, and length of follow-up. Similarly, our finding of an inconsistent relationship between self-reported history of diabetes and hypertension in older adults in Africa in the current review contrasts with that in our previous review in which it was a consistent determinant of hypertension in West African workers [11].

Some limitations of this review should be noted. A major limitation was the limited number of covariates, particularly lifestyle and comorbid factors, investigated in individual studies for their relationship with hypertension. It is therefore likely that some important confounders were not included in the models. Even in those where several covariates were included in the models presented, there are likely to still be unmeasured confounders. The effect sizes from the few studies which enrolled non-representative samples may be biased. The study populations were diverse and so the study findings may not be generalizable to specific groups. The differences in ability of participants to recall history of past illness or behaviour could explain differences in the relationships observed between studies. The definition of some lifestyle covariates such as alcohol consumption and physical activity differed between studies making comparisons difficult. The statistical determinants identified in this review do not imply causality, especially because most of the included studies were cross-sectional. For example, the consistent association between a history of stroke and having hypertension should not be interpreted to mean that stroke is a biological risk factor for hypertension.

## Conclusions

We identified older age, overweight/obesity, female sex, urban residence, history of stroke as the frequent or consistent determinants of systemic hypertension in older adults in Africa. Socioeconomic and lifestyle factors generally had variable relationships with hypertension. Further research with longitudinal studies is needed to better define the determinants of incident hypertension in this group. Future reviews could perform meta-analysis to obtain pooled estimates of the risk factors as well as explore what strategies such as weight reduction throughout the life course, including the middle and older age groups, improve cardiovascular health so that public health authorities can implement the most appropriate policies.

## Additional files

Additional file 1: **Table S1.** Search strategy. (DOCX 14 kb)

Additional file 2: **Table S2.** Evaluation of risk of bias from primary studies on hypertension in older adults in Africa. (DOCX 16 kb)

## Data Availability

The datasets generated and/or analyzed during the current study are available in the repository: 10.17635/lancaster/researchdata/267.

## Abbreviations

BMI: Body mass index
CAR: Central African Republic;
DHTN: Diastolic Hypertensio
HTN: Hypertension
NCD: Noncommunicable diseases
PRISMA: Preferred reporting items for systematic reviews and meta-analyses
SAGE: Study on global ageing and adult health
STHN: Systolic hypertension
