# Supplementary material for: Determinants of systemic hypertension in older adults in Africa: a systematic review
Source: BMC Cardiovasc Disord. 2019 Jul 22;19:173. doi: 10.1186/s12872-019-1147-7 (PMC6647089; doi:10.1186/s12872-019-1147-7)
Supplement: Supplementary file 1 — Table S1. Search strategy. (DOCX 14 kb) [file 12872_2019_1147_MOESM1_ESM.docx]

## Table S1. Search strategy

| Search # | Search words |
| --- | --- |
| 1 | exp hypertension/ep, et, pc [Epidemiology, Etiology, Prevention] |
| 2 | (Hypertensi* or "blood pressure" or cardiovascular or cardiometabolic).ab. |
| 3 | 1 or 2 |
| 4 | exp prevalence/ |
| 5 | exp incidence/ |
| 6 | (Prevalence or proportion or survey or descriptive or cross-sectional or cohort or longitudinal or "attributable fraction" or incidence).ab. |
| 7 | (Prevalence or proportion or survey or descriptive or cross-sectional or cohort or longitudinal or "attributable fraction" or incidence).sh. |
| 8 | 4 or 5 or 7 |
| 9 | exp Africa/ |
| 10 | indian ocean/ or exp mauritius/ or exp reunion/ or exp seychelles/ |
| 11 | (africa* or algeria* or angola* or benin* or botswana* or burkina* or burundi* or cameroon* or canary islands or cabo verde or cape verde* or central african republic or chad or comoros or comores or congo* or democratic republic of the congo or djibouti or egypt* or equatorial guinea* or eritrea* or ethiopia* or gabon* or gambia* or ghana* or guinea* or bissau or cote d'ivoire or kenya* or lesotho or liberia* or libya* or jamahiriya or jamahiryia or madagascar or malawi* or mali* or mauritania* or mauritius or morocc* or mozambi* or mocambique or namibia* or niger* or nigeria* or reunion or rwanda* or sao tome or senegal* or seychelles or sierra leone* or somalia* or south africa* or st helena or sudan* or swazi* or tanzania* or tanganyika or togo* or tunisia* or uganda* or western sahara or zaire* or zambia* or zimbabwe*).sh. |
| 12 | 9 or 10 or 11 |
| 13 | 3 and 8 and 12 |
| 14 | limit 13 to ("middle age (45 to 64 years)" or "all aged (65 and over)") |
| 15 | limit 14 to humans |
