# Supplementary material for: Determinants of systemic hypertension in older adults in Africa: a systematic review
Source: BMC Cardiovasc Disord. 2019 Jul 22;19:173. doi: 10.1186/s12872-019-1147-7 (PMC6647089; doi:10.1186/s12872-019-1147-7)
Supplement: Supplementary file 2 — Table S2. Evaluation of risk of bias from primary studies on hypertension in older adults in Africa. (DOCX 16 kb) [file 12872_2019_1147_MOESM2_ESM.docx]

Table S2 Evaluation of the risk of bias in included primary studies on the determinants of hypertension

| No. | Primary Reference | *Q1* | *Q2* | *Q3* | *Q4* | *Q5* | *Q6* | *Q7* | *Q8* | *Q9* | *Q10* | *Overall risk of study bias* |
| --- | --- | --- | --- | --- | --- | --- | --- | --- | --- | --- | --- | --- |
| 1 | Abegunde 2013 | Low risk | Low risk | Low risk | Low risk | High risk | Low risk | Low risk | Low risk | Low risk | Low risk | **Low risk** |
| 2 | Dewhurst 2013 | Low risk | Low risk | Low risk | Low risk | Low risk | Low risk | Low risk | Low risk | Low risk | Low risk | **Low risk** |
| 3 | Guerchet 2012A | Low risk | Low risk | Low risk | Low risk | Low risk | Low risk | Low risk | Low risk | Low risk | Low risk | **Low risk** |
| 4 | Hammami 2011 | High risk | Low risk | Low risk | Low risk | Low risk | Low risk | Low risk | Low risk | Low risk | Low risk | **Low risk** |
| 5 | Hien 2014 | Low risk | Low risk | Low risk | Low risk | Low risk | Low risk | Low risk | Low risk | Low risk | Low risk | **Low risk** |
| 7 | Koopman 2012 | Low risk | Low risk | Low risk | Low risk | Low risk | Low risk | Low risk | Low risk | Low risk | Low risk | **Low risk** |
| 9 | Mathenge 2010 | Low risk | Low risk | Low risk | Low risk | Low risk | Low risk | Low risk | Low risk | Low risk | Low risk | **Low risk** |
| 10 | Minicuci 2014 | Low risk | Low risk | Low risk | Low risk | Low risk | Low risk | Low risk | Low risk | Low risk | Low risk | **Low risk** |
| 11 | Mkhize 2013 | Low risk | Low risk | Low risk | Low risk | Low risk | Low risk | Low risk | Low risk | Low risk | Low risk | **Low risk** |
| 16 | Pilleron 2017A | Low risk | Low risk | Low risk | Low risk | Low risk | Low risk | Low risk | Low risk | Low risk | Low risk | **Low risk** |
| 18 | Scholten 2011 | Low risk | Low risk | Low risk | Low risk | Low risk | Low risk | Low risk | Low risk | Low risk | Low risk | **Low risk** |
| 19 | Tianyi 2017 | Low risk | Low risk | Low risk | Low risk | Low risk | Low risk | Low risk | Low risk | Low risk | Low risk | **Low risk** |

List of the 10 questions (Q1 – Q10) applied to the studies:

1. *Was the study's target population a close representation of the national population in relation to relevant variables, e.g. age, sex, occupation?*
2. *Was the sampling frame a true or close representation of the target population?*
3. *Was some form of random selection used to select the sample, OR, was a census undertaken?*
4. *Was the likelihood of non-response bias minimal?*
5. *Were data collected directly from the subjects (as opposed to a proxy)?*
6. *Was an acceptable case definition used in the study?*
7. *Was the study instrument that measured the parameter of interest (e.g. prevalence of low back pain) shown to have reliability and validity (if necessary)?*
8. *Was the same mode of data collection used for all subjects?*
9. *Was the length of the shortest prevalence period for the parameter of interest appropriate?*
10. *Were the numerator(s) and denominator(s) for the parameter of interest appropriate?*
